# Non-Alcoholic Fatty Liver Disease (NAFLD) and risk of new-onset heart failure: a retrospective analysis of 173,966 patients

**DOI:** 10.1007/s00392-023-02250-z

**Published:** 2023-07-06

**Authors:** Christoph Roderburg, Sarah Krieg, Andreas Krieg, Sascha Vaghiri, Raphael Mohr, Marcel Konrad, Mark Luedde, Tom Luedde, Karel Kostev, Sven H. Loosen

**Affiliations:** 1 Department of Gastroenterology, Hepatology and Infectious Diseases, University Hospital Duesseldorf, Medical Faculty of Heinrich Heine University Duesseldorf, Moorenstraße 5, 40225 Duesseldorf, Germany; 2 Department of Surgery (A), University Hospital Duesseldorf, Medical Faculty of Heinrich Heine University Duesseldorf, 40225 Duesseldorf, Germany; 3 Department of Hepatology and Gastroenterology, Charité University Medicine Berlin, 13353 Berlin, Germany; 4 FOM University of Applied, Sciences for Economics and Management, 60549 Frankfurt Am Main, Germany; 5 Christian-Albrechts-University of Kiel, 24118 Kiel, Germany; 6 Epidemiology, IQVIA, 60549 Frankfurt, Germany

**Keywords:** NAFLD, Non-alcoholic fatty liver disease, NASH, Non-alcoholic steatohepatitis, Heart failure, Epidemiology, Prevention

## Abstract

**Background:**

Non-alcoholic fatty liver disease (NAFLD) represents the leading cause of chronic liver disease. Its high mortality and morbidity are mainly caused by non-hepatic comorbidities and their clinical complications. Accumulating evidence suggests an association between NAFLD and heart failure (HF), but large-scale data analyses from Germany are scarce.

**Methods:**

Using the Disease Analyzer database (IQVIA), this analysis retrospectively evaluated two cohorts of outpatients with and without NAFLD with respect to the cumulative incidence of HF as the primary outcome between January 2005 and December 2020. Cohorts were propensity score matched for sex, age, index year, yearly consultation frequency, and known risk factors for HF.

**Results:**

A total of 173,966 patients were included in the analysis. Within 10 years of the index date, 13.2% vs. 10.0% of patients with and without NAFLD were newly diagnosed with HF (*p* < 0.001). This finding was supported by univariate Cox regression analysis in which NAFLD was found to be significantly associated with subsequent HF (Hazard Ratio (HR) 1.34, 95% Confidence Interval (CI) 1.28–1.39, *p* < 0.001). The association between NAFLD and HF was observed across all analysed age groups and as comparable between both men (HR 1.30, 95% CI 1.23–1.38; *p* < 0.001) and women (HR: 1.37, 95% CI 1.29–1.45; *p* < 0.001).

**Conclusion:**

NAFLD is significantly associated with an increased cumulative incidence of HF, which, given its rapidly increasing global prevalence, could be crucial to further reduce its high mortality and morbidity. We recommend risk stratification within a multidisciplinary approach for NAFLD patients, including systematic prevention or early detection strategies for HF.

**Supplementary Information:**

The online version contains supplementary material available at 10.1007/s00392-023-02250-z.

## Introduction

Non-alcoholic fatty liver disease (NAFLD) is a collective term for a broad spectrum of liver diseases ranging from simple steatosis (non-alcoholic fatty liver, NAFL) to non-alcoholic steatohepatitis (NASH) and its complications fibrosis, cirrhosis and hepatocellular carcinoma [1, 2]. With a prevalence of 25–30%, NAFLD has become the most common cause of chronic liver disease in the Western world [3–5] and is expected to increase dramatically in the future with the growing epidemics of obesity and type 2 diabetes (T2DM) [5].

It is associated with increased all-cause mortality, less due to liver complications than to non-hepatic comorbidities and their complications [6, 7], with cardiovascular events being the most common cause of death in NAFLD [8, 9]. In this context, NAFLD has been related to an increased risk of developing coronary heart disease, T2DM, chronic kidney disease and certain extrahepatic cancers [10–12]. More recently, accumulating evidence suggests a strong and independent association between NAFLD and an increased risk of functional, structural and arrhythmic cardiac complications that promote the development of heart failure (HF) [8–10, 13]. While the mechanisms linking NAFLD and HF remain largely unknown [10], NAFLD and HF share many risk factors, including T2DM, obesity, hypertension, and metabolic syndrome [10, 14, 15]. Although several studies have found an association between NAFLD and HF, the results are sometimes conflicting, and most studies had small samples or limited populations or did not adequately adjust for potential confounders [10, 16, 17].

As both NAFLD and HF are associated with high morbidity and mortality worldwide and thus represent a major economic and public health burden, the aim of this work was to investigate the incidence of HF as a function of NAFLD as the primary outcome after adjustment for HF risk factors in a large real-world cohort from Germany.

### Methods

## Database

The present study used data from the Disease Analyzer (DA) database (IQVIA). This database has already been extensively described in the literature [18]. To summarize, the DA database includes data on demographic variables, diagnoses, and prescriptions obtained in general and specialized practices in Germany. The quality of the data is assessed every month based on several criteria (e.g., completeness of documentation and linkage between diagnoses and prescriptions). Practices to include in the database are selected according to the yearly statistics of the German Medical Association, which include information on physician’s age, specialty group, community size category, and German federal state. Finally, it has been shown in prior research that the DA database is representative of all practices in Germany [18].

### Study population

**Fig. 1:**
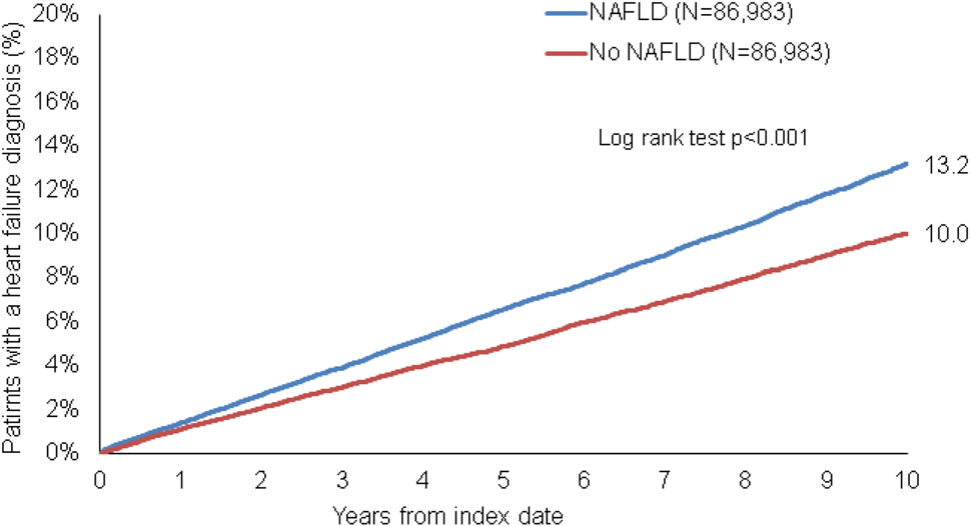
Kaplan–Meier curves for Time to Heart Failure Diagnosis in Patients with and without NAFLD

This retrospective cohort study included adult patients (≥ 18 years) with an initial diagnosis of NAFLD (ICD-10: K75.8, K76.0) in 1,262 general practices (GP) in Germany between January 2005 and December 2020 (index date; Fig. 1). Further inclusion criterium was an observation time of at least 12 months prior to the index date as well as at least one documented body mass index (BMI) within six months prior to or at the index date. Patients with other liver disorders (ICD-10: B18, K70-K77), and heart failure (ICD-10: I50) diagnoses prior to or on the index date were excluded.

NAFLD patients were matched to non-NAFLD individuals by propensity scores based on sex, age, index year, yearly consultation frequency and BMI at baseline. As NAFLD patients have much higher consultation frequency by GPs, and higher consultation frequency can increase the probability of other diagnoses documentation, we included consultation frequency per year in the matching. BMI was included as obesity is strongly associated with both NAFLD and T2DM. For the non-NAFLD individuals, the index date was that of a randomly selected visit between January 2005 and December 2020.

### Study outcomes and statistical analyses

The main outcome of the study was the cumulative incidence of HF (ICD 10: I50) as a function of NAFLD. Differences in the sample characteristics between those with and those without NAFLD were compared using the Wilcoxon signed-rank test for continuous age, the Stuart-Maxwell test for categorical age, and the McNemar test for sex and comorbidities. Univariable Cox regression models were performed to study the association between NAFLD and HF. These models were performed separately for women, men, four age groups, and patients with and without diabetes at baseline.

In addition, we conducted several sensitivity analyses. First, Cox regression was adjusted for chronic kidney disease documented within 12 months prior to or at the index date. Second, Cox regression was adjusted for antihypertensive and statin therapy prescribed within 12 months before or at the index date. Third, only patients without a history of myocardial infarction or dilatative cardiomyopathy prior to the index date were included. Fourth, a regression analysis was performed counting only incident HF events that occurred at least three years after NAFLD diagnosis. To counteract the problem of multiple comparisons, *p*-values < 0.01 were considered statistically significant. Values between 0.01 and < 0.05 were considered statistical tendency. Analyses were carried out using SAS version 9.4 (SAS Institute, Cary, USA).

## Results

### Characteristics of the study cohort

A total of 86,983 patients diagnosed with NAFLD between January 2005 and December 2020 in 1,262 GPs in Germany were selected for this analysis and compared with a propensity score-matched cohort of 86,983 patients without NAFLD. The mean age of the study participants was 57.2 years with a standard deviation (SD) of 14.3 years. Among the patients, 52.6% were male. The mean yearly consultation frequency for NAFLD and non-NAFLD patients was 8.6 visits per year. The mean BMI was 29.2 kg/m^2^ for NAFLD patients and 29.1 kg/m^2^ for non-NAFLD patients, with no significant difference between the two groups (*p* = 0.209). Regarding relevant comorbidities, 27.1% of patients suffering from diabetes, 19.3% from obesity, 55.7% of patients were diagnosed with hypertension, 15.3% with ischemic heart disease and 2.4% with atrial fibrillation, respectively. 10.3% of the patients displayed chronic obstructive pulmonary disease (COPD). No differences were found between the NAFLD and non-NAFLD patients in terms of these variables analyzed in this study. At baseline, statins and antihypertensive drugs (diuretics, beta-blockers, calcium channel blockers, angiotensin-converting enzyme (ACE) inhibitors, angiotensin receptor blockers (ARBs)) were slightly less commonly prescribed in the NAFLD cohort than in the non-NAFLD cohort. The patient characteristics are detailed in Table 1.

**Table 1:** Basic Characteristics of the Study Sample after 1:1 Matching

Variables included in Matching	Proportion affected among patients with NAFLD (%) *N* = 86,983	Proportion affected among patients without NAFLD (%) *N* = 86,983	*p*-value
Variable			
Age (Mean, SD)	57.2 (14.3)	57.2 (14.3)	0.979
Age 18–50	30.8	31.0	0.682
Age 51–60	29.7	26.6	
Age 61–70	23.5	23.4	
Age > 70	19.0	19.0	
Women	47.4	47.4	1.000
Men	52.6	52.6	
Yearly consultation frequency (Mean, SD)	8.6 (6.4)	8.6 (6.4)	0.996
Diabetes	27.1	27.1	0.953
Obesity	19.3	19.3	0.995
Hypertension	55.7	55.7	0.958
COPD	10.3	10.3	0.994
Ischemic heart disease	15.3	15.3	0.947
Atrial fibrillation	2.4	2.4	0.887
Further variables documented within 12 months prior to or at the index date			
Chronic kidney disease	6.8	5.1	< 0.001
Prescriptions of statins	15.4	16.2	< 0.001
Prescriptions of diuretics	8.4	10.3	< 0.001
Prescriptions of betablockers	20.8	23.5	< 0.001
Prescriptions of calcium channel blockers	11.0	12.4	< 0.001
Prescriptions of ACE inhibitors	14.4	17.0	< 0.001
Prescriptions of ARB	14.8	15.1	0.084

Proportions of patients given in %, unless otherwise indicated. *SD* standard deviation

### NAFLD is associated with a higher risk of developing HF

During a 10-year follow-up period, 13.2% of patients diagnosed with NAFLD and 10.0% of patients without NAFLD developed HF (as shown in Fig. 1, *p* < 0.001). This finding was confirmed by univariate Cox regression analysis, which showed a significant positive association between NAFLD and subsequent HF diagnosis (Hazard Ratio (HR) 1.34, 95% Confidence Interval (CI) 1.28–1.39; *p* < 0.001). Interestingly, the proportion of patients who experienced a first myocardial infarction (1.7% vs. 1.5%, *p* = 0.004) or atrial fibrillation (6.0% vs. 4.6%, *p* < 0.001) between the index date and the end of follow-up was higher in the NAFLD cohort than in the non-NAFLD cohort. This strong association between NAFLD and HF persisted in all age groups analyzed, but was particularly evident in patients with NAFLD between the ages of 18–50 years (HR 1.62, 95% CI 1.39–1.89; *p* < 0.001) and markedly weaker in older patients. Notably, the effect of NAFLD on HF was found to be similar in men (HR 1.30, 95% CI 1.23–1.38; *p* < 0.001) and women (HR 1.37, 95% CI 1.29–1.45; *p* < 0.001) with NAFLD and in patients with (HR 1.30, 95% CI 1.21–1.40; *p* < 0.001) and without diabetes (HR 1.35, 95% CI 1.30–1.38; *p* < 0.001) (Table 2). Sensitivity analyses confirmed the results of the univariate analyses. The positive association between NAFLD and subsequent HF was significant after adjustment for chronic kidney disease (HR 1.29, 95% CI 1.24–1.34; *p* < 0.001) or after adjustment for antihypertensive and statin therapy (HR 1.41, 95% CI 1.36–1.47; *p* < 0. 001), when including only patients without a history of myocardial infarction or dilatative cardiomyopathy before the index date (HR 1.44, 95% CI 1.38–1.50; *p* < 0.001), and when counting incident HF events after at least three years of NAFLD (HR 1.30, 95% CI 1.23–1.37; *p* < 0.001) (Table 3).

**Table 2:** Association between NAFLD and the Incident Heart Failure Diagnoses in Patients followed in General Practices in Germany (Cox Regression Models)

Cohort	Hazard Ratio (95% CI)	*p*-value
Total	1.34 (1.28–1.39)	< 0.001
Age 18–50	1.62 (1.39–1.89)	< 0.001
Age 51–60	1.35 (1.22–1.49)	< 0.001
Age 61–70	1.36 (1.26–1.47)	< 0.001
Age > 70	1.22 (1.15–1.30)	< 0.001
Women	1.37 (1.29–1.45)	< 0.001
Men	1.30 (1.23–1.38)	< 0.001
Diabetes	1.30 (1.21–1.40)	< 0.001
No diabetes	1.35 (1.30–1.38)	< 0.001

**Table 3:** Sensitivity analyses for the Association between NAFLD and the Incident Heart Failure Diagnoses in Patients followed in General Practices in Germany (Cox Regression Models)

Cohort	Hazard Ratio (95% CI)	*p*-value
Adjusted for chronic kidney disease	1.29 (1.24–1.34)	< 0.001
Adjusted for antihypertensive and statin therapy prescribed within 12 months prior to or at the index date	1.41 (1.36–1.47)	< 0.001
Only patients without a history of myocardial infarction or dilatative cardiomyopathy prior to the index date	1.44 (1.38–1.50)	< 0.001
Incident HF event after at least three years of NAFLD	1.30 (1.23–1.37)	< 0.001

## Discussion

This retrospective study using the DA database (IQVIA) included a large real-world cohort of more than 173,966 adult outpatients in Germany and compared individuals with and without NAFLD in a 1:1 matched cohort for the risk of new-onset HF as the primary outcome. It was found that NAFLD was associated with a significantly increased risk of developing HF in all age groups, in both sexes and in patients with and without diabetes at baseline. As such, during the 10-year follow-up period, 13.2% of patients with NAFLD were newly diagnosed with HF compared to 10.0% of patients without NAFLD. Notably, the comparison of patients with and without NAFLD was adjusted for sex, age, and diagnoses considered risk factors for HF, such as obesity, diabetes, hypertension, ischemic heart disease, atrial fibrillation, chronic kidney disease and chronic obstructive pulmonary disease (COPD).

Our study supports the findings of a recently published large meta-analysis by Li et al. which included a total of 6 cohort studies with approximately 11 million participants and found a 36% increased relative risk of future HF in patients with NAFLD compared with patients without NAFLD after adjustment for other cardiometabolic risk factors. Of interest, even simple steatosis was associated with an increased risk of HF [16]. Similarly, in an updated meta-analysis of 11 observational cohort studies with aggregated data from about 11 million middle-aged individuals from various countries, Mantovani et al. examined new-onset HF as the primary outcome in relation to NAFLD over a median of 10 years. The authors found that NAFLD was significantly associated with a 1.5-fold long-term risk of new-onset HF that was independent of age, sex, obesity, diabetes, hypertension, and other common cardiovascular risk factors [19]. Of note, in our study, although a significant association between NAFLD and incident HF was found in all age groups studied, interestingly, this association was particularly pronounced in patients with NAFLD aged 18 to 50 years, whereas it was weaker in older patients. Possible hypotheses that could at least partially explain this observation are, first, that older age, which is a strong risk factor for HF, may attenuate the effect of NAFLD itself as a risk factor for new-onset HF and, second, that older people are more likely to be treated with medication, which in turn may reduce the risk of cardiovascular disease and the development of HF. Further research, particularly with prospective study designs, is needed to better understand this age-related association.

The development of HF is a process of cardiac remodeling based on molecular, cellular, and interstitial changes in the heart induced by hemodynamic stress, neurohormonal activation, and other factors yet to be elucidated, which manifests clinically as alterations in cardiac structure and function [20]. In this regard, several studies have found a strong association between NAFLD and an increased risk of left ventricular (LV) diastolic dysfunction, LV hypertrophy, or left atrial enlargement independent of obesity, hypertension, and T2DM [9, 21].

Recently, a meta-analysis of 16 cross-sectional studies involving 32,000 subjects showed that NAFLD is associated with subclinical changes in left heart structure and function. Interestingly, the association between NAFLD and subclinical cardiac remodeling remained significant in most of the studies included in the meta-analysis when adjusted for established cardiometabolic risk factors [21]. In addition, Chiu et al. examined a Framingham Heart Study sample of 2,356 participants who had undergone echocardiography and standardized computed tomography (CT) to measure liver fat [22]. They demonstrated that liver fat was associated with several imaging markers of subclinical cardiac dysfunction after correction for a number of demographic and HF risk factors. Similar conclusions were reached in the population-based cohort study CARDIA (Coronary Artery Risk Development in Young Adults), which included 1827 adults and prospectively examined whether NAFLD was linked to short-term changes in echocardiographic measures of LV structure and function over a 5-year period. Again, NAFLD was found to be associated with subclinical changes in LV structure and function independent of traditional risk factors for HF [23].

While the association between NAFLD and HF risk remains largely unclear, several potential pathophysiological mechanisms by which NAFLD may increase the risk of HF development and progression have been discussed [10, 19]. Although our study did not elucidate the mechanisms linking NAFLD to HF, it suggests that NAFLD, beyond its phenotype, is a systemic disease whose pathophysiology is determined by the involvement of multiple organ systems. These mechanisms are thought to be determined by a variety of metabolic, genetic, epigenetic, and nutritional factors [19]. In particular, factors associated with T2DM and obesity have been discussed as a possible link between NAFLD and the development of HF [24]. Thus, insulin resistance and impaired glucose and lipid metabolism, which are among the major pathophysiological features of NAFLD, are thought to play a role in alterations in cardiac energy metabolism and cardiac dysfunction [10]. In this context, circulating free fatty acids (FFAs) and triglycerides have been reported to lead to increased myocardial fatty acid oxidation and inefficient metabolism in both liver and cardiac myocytes in patients with NAFLD, which may contribute to the development of HF [10, 20]. In addition, a role for atherogenic dyslipidemia, particularly in mediating NAFLD-associated cardiovascular risk through an increased hepatic synthesis of very low-density lipoprotein (VLDL) in NAFLD, has been described [10]. Furthermore, hepatic and cardiac mitochondrial dysfunction has been linked to the development of progressive NAFLD [9, 11, 25, 26], which in turn leads to increased production of reactive oxygen species (ROS) [27]. There is also evidence that increased activation of the renin–angiotensin–aldosterone system (RAAS), known to be an important mediator of cardiac remodeling and progression of HF, may play an additional role in NAFLD [28]. In this context, an increased activation of the RAAS in combination with cardiac ROS is thought to be involved in the interaction between NAFLD and HF [10, 28]. It has also been suggested that the proinflammatory cytokines interleukin-6 (IL-6), interleukin-1beta (IL-1β), and tumor necrosis factor-alpha (TNF-α), as well as decreased plasma adiponectin concentrations resulting from increased or dysfunctional visceral adipose tissue, may not only be involved in the development and progression of NAFLD through effects on glucose and lipid metabolism and insulin resistance but may also affect the coronary arteries and cause HF [29, 30]. Recently, intestinal dysbiosis has received increasing attention as a factor linking NAFLD and HF. In this context, gastrointestinal factors such as the endotoxin lipopolysaccharide (LPS), metabolites of aromatic acids, ethanol, short-chain fatty acids, incretins, and modified bile acids are known to contribute to the severity of liver disease [31, 32] and may also have direct effects on the cardiovascular system, potentially influencing HF [33]. Increased intestinal permeability may also enhance the potential of LPS to enter the portal circulation and promote a proinflammatory response in the liver. In this regard, trimethylamine oxide (TMAO) has also been discussed as having a potential adverse effect on cardiac disease [33, 34]. Also of interest are genetic variants, such as the presence of gene polymorphisms, e.g. for patatin-like phospholipase domain-containing protein-3 (PNPLA3) and trans-membrane 6 superfamily 2 (TM6SF2) [10, 19, 35], which have been implicated in predisposing to the development and severity of liver disease in NAFLD [35–37]. Although both genotypes increase the risk of more severe liver disease in NAFLD, there is evidence that both genotypes also decrease plasma concentrations of VLDL, which may attenuate the strength of the association between NAFLD and the risk of coronary heart disease in particular [9]. However, to our knowledge, no study has examined whether and to what extent these genetic polymorphisms influence the strength of the association between NAFLD and the risk of heart failure, and further evidence is needed.

To date, no specific pharmacotherapy has been approved for NAFLD, and treatment of NAFLD is primarily based on lifestyle changes (e.g., weight reduction, physical activity) [38]. However, there is evidence that certain medications used to treat T2DM may also reduce the risk of HF in patients with NAFLD. In this context, scientific interest in the relationship between these two conditions is increasing as newer antihyperglycemic agents, particularly sodium-glucose cotransporter-2 (SGLT2) inhibitors and glucagon-like peptide-1 receptor (GLP1R) agonists, have shown some benefit on liver fat content and histologic regression of NASH [39], but also clinically significant benefits on cardiovascular outcomes, including the risk of hospitalization for HF, independent of T2DM status [39, 40].

We would like to point out some limitations of our study, which are mainly due to the study design and are therefore unavoidable. First, our study is subject to the limitations of a retrospective analysis of a database. Secondary data analyses may be limited by the incompleteness of the underlying data. Because all diagnoses are based on ICD-10 codes, misclassification and undercoding of certain diagnoses cannot be excluded, which may have resulted in some selection bias. In addition, certain information about important health determinants that would have allowed further analysis was not available, such as socioeconomic status, family history, ethnic background/race, environmental conditions, lifestyle factors (e.g., physical activity, nicotine or alcohol consumption, diet), and genetic information. Another limitation of our study is that the DA database does not yet include imaging (including sonography/elastography/fibroscan and echocardiography) or histopathologic data in addition to ICD-10 codes, and it does not include clinical follow-up information that would have allowed more accurate stratification of NAFLD and HF. In particular, it would have been interesting to investigate whether the severity of liver disease in NAFLD would have further exacerbated the increased risk of developing HF. At this point, we would like to refer to previously published cohort studies using liver biopsy or non-invasive scores for advanced fibrosis, showing that the risk of developing HF seems to further increase with the severity of NAFLD, especially with higher fibrosis stages [19, 41, 42]. As mentioned above, because echocardiographic data were not available for our analyses, we were also unable to examine the association between NAFLD and HF as a function of left ventricular ejection fraction (HF with preserved ejection fraction (HFpEF) versus HF with reduced ejection fraction (HFrEF)). Furtherrmore, we should be aware that our study is purely descriptive and can only show associations, but cannot draw any causal conclusions. Nevertheless, we would like to emphasize the strengths of our study, which are the large number of patients included, the use of representative population data, and the overall long follow-up period. Our observations in a large real-world cohort in Germany are of particular importance for the generalizability of the results of previously published cohort studies to Western countries. Moreover, this study used data from the representative IQVIA DA database, which has been validated in several studies [18] and has already been used in studies on NAFLD [43, 44] and HF [45, 46].

In conclusion, the present study provides strong evidence for a positive association between NAFLD and subsequent HF. Given the dramatic increase in the prevalence of NAFLD and the high morbidity and mortality of HF, the results of our study are of major clinical relevance. Therefore, we propose a multidisciplinary approach that includes prevention and screening strategies for further patient-centered risk stratification using echocardiography or serum biomarkers to identify the early stages of HF in patients with NAFLD. The results of our study should stimulate further research efforts to understand the complex pathophysiological mechanisms by which NAFLD may contribute to the risk of new-onset HF and to determine whether amelioration of NAFLD may prevent or slow the development and progression of HF.

### Supplementary Information

Below is the link to the electronic supplementary material.

Supplementary file1 (DOCX 14 kb)

## Data Availability

The data supporting the results of this study are available from the corresponding author upon reasonable request.
