# Supplementary material for: Non-Alcoholic Fatty Liver Disease (NAFLD) and risk of new-onset heart failure: a retrospective analysis of 173,966 patients
Source: Clin Res Cardiol. 2023 Jul 6;112(10):1446–53. doi: 10.1007/s00392-023-02250-z (PMC10562311; doi:10.1007/s00392-023-02250-z)
Supplement: Supplementary file 1 — Supplementary file1 (DOCX 14 kb) [file 392_2023_2250_MOESM1_ESM.docx]

**Suppl. Table 1.** Basic Characteristics of the Study Sample by Heart Failure Event

| Variable | Proportion affected among patients  with Heart Failure (%)  N=10,516 | Proportion affected among patients  without Heart Failure (%)  N=163,450 | p-value |
| --- | --- | --- | --- |
| Age (Mean, SD) | 67.5 (11.0) | 56.9 (8.4) | <0.001 |
| Age 18-50 | 7.8 | 31.0 | <0.001 |
| Age 51-60 | 17.3 | 28.1 |  |
| Age 61-70 | 30.2 | 23.6 |  |
| Age >70 | 44.7 | 17.3 |  |
| Women | 48.8 | 46.4 | <0.001 |
| Men | 51.2 | 53.6 |  |
| Yearly consultation frequency (Mean, SD) | 12.0 (7.3) | 8.4 (6.0) | <0.001 |
| Diabetes | 39.5 | 23.1 | <0.001 |
| Obesity | 22.4 | 21.0 | 0.158 |
| Hypertension | 77.9 | 55.3 | <0.001 |
| COPD | 14.2 | 9.6 | <0.001 |
| Ischemic heart disease | 26.7 | 10.5 | <0.001 |
| Atrial fibrillation | 7.4 | 2.0 | <0.001 |
| Chronic kidney disease | 12.3 | 5.5 | <0.001 |
| Prescriptions of statins | 26.7 | 15.1 | <0.001 |
| Prescriptions of diuretics | 22.1 | 8.5 | <0.001 |
| Prescriptions of betablockers | 37.5 | 21.1 | <0.001 |
| Prescriptions of calcium channel blockers | 20.9 | 11.1 | <0.001 |
| Prescriptions of ACE inhibitors | 23.4 | 15.2 | <0.001 |
| Prescriptions of ARB | 21.7 | 14.5 | <0.001 |

Proportions of patients given in %, unless otherwise indicated. SD: standard deviation.
